# Age and *Staphylococcus aureus* Inoculation Route Differentially Alter Metabolic Potential and Immune Cell Populations in Laying Hens

**DOI:** 10.3389/fvets.2021.653129

**Published:** 2021-03-26

**Authors:** Krysten Fries-Craft, Meaghan M. Meyer, Yuko Sato, Mohamed El-Gazzar, Elizabeth A. Bobeck

**Affiliations:** ^1^Department of Animal Science, Iowa State University, Ames, IA, United States; ^2^Department of Veterinary Diagnostic and Production Animal Medicine, Iowa State University, Ames, IA, United States

**Keywords:** poultry, *Staphylococcus aureus*, immunity, flow cytometry, Seahorse metabolic assay

## Abstract

In 2018 and 2019, *Staphylococcus aureus* was isolated from multiple post-molt commercial laying hens with unusually high mortality. A challenge study was conducted to elucidate the role of *S. aureus* in this disease outbreak and the work herein represents the assessment of immunological responses in laying hens experimentally infected with *S. aureus* isolates from these cases. A total of 200 laying hens at 22 or 96 weeks of age (100/ age group) were assigned to 1 of 4 experimental inoculation groups (negative control, oral gavage, subcutaneous injection, or intravenous injection) after a 72 h acclimation period. Blood samples were taken prior to inoculation (baseline), 6 h post-inoculation (pi), 24 hpi, 3 dpi, and 7 dpi. Additional spleen samples to further assess systemic immunity were taken at baseline, 3 and 8 dpi. Metabolic phenotypes of peripheral blood mononuclear cells (PBMC) were isolated and assessed by Seahorse metabolic assay. Immune cell profiles in the spleen and PBMC were assessed by multicolor flow cytometry. At baseline, 96-week-old laying hens had 26.7% fewer PBMC-derived T cells compared to 22-week-old birds. Older hens had 28.9% increased helper T cell (T_H_) populations and 60.5% reduced γδ T cells (*P* = 0.03 and < 0.0001) which may contribute to variable clinical responses between age groups; however, no age-related differences in metabolic potential were observed. Metabolic outcomes showed that birds remained stressed from transport and re-housing past a 72 h acclimation period and through 24 h- 3 days post-inoculation. Inoculation with *S. aureus* generally reduced oxidative and glycolytic potentials compared to the control, with the greatest reductions observed in birds inoculated by intravenous injection (*P* < 0.05). Overall CD3^+^ T cell populations showed significant reductions in the intravenous group compared to other inoculation routes from 24 hpi to 7 dpi (23.6–39.0%; *P* ≤ 0.0001). These results suggest that age-related baseline differences in T cell populations and changes to T cell subpopulations and other immune cells due to inoculation route may have an additive effect on *S. aureus*- induced reductions in metabolic potential; however, further research linking metabolic potential and immune cell profiles is needed.

## Introduction

*Staphylococcus aureus* is part of the skin and gastrointestinal tract microbiota of healthy humans and animals but is also associated with secondary infections as an opportunistic pathogen (1). During the fall and spring of 2018 and 2019, high mortality events in multiple post-molt layer flocks presenting with swollen combs and wattles, fever, and reduction in egg production from one commercial multi-age complex were attributed to an apparent systemic bacterial infection (2). *Staphylococcus aureus* was the main bacterial isolate from multiple internal organs including spleen and bone marrow (2). In poultry, *S. aureus* does not typically cause a systemic infection contributing to septicemia and high mortality and there is only one previous report of a similar outbreak in laying hens from 1974 (3). Underlying age-related immune suppression could be a predisposing factor that may explain this unusual clinical presentation that affected post-molt layers and not younger hens within the same complex. In this work, we examined interactions between the immune system and *S. aureus* as part of a larger challenge study to elucidate the role of *S. aureus* in these unusual outbreaks (2).

Laying hens from two age groups (22 and 96 weeks old) were challenged with *S. aureus* isolated from the described naturally-occurring outbreak *via* three different routes in an attempt to recreate this unusual clinical presentation. It was previously reported that *S. aureus* inhibits phagocyte killing functions and their ability to migrate into affected tissues to evade an immune response during infection (4–6). Additionally, *S. aureus* alters T cell activity during the adaptive response through direct T cell lysis or superantigen-mediated activation, which causes deleterious cytokine production associated with toxic shock syndrome in humans (7–9). Recent assessment of specific immune responses to *S. aureus* and impacts on cellular metabolic profiles in poultry is limited and focuses primarily on humoral responses for vaccine development (10). The objective was to evaluate changes to immunometabolism and systemic immune cell profiles in 22- and 96-week-old laying hens inoculated with *S. aureus* isolates by various routes. This will provide better understanding as to which aspects of the immune system are involved in responding to such systemic bacterial infections. In turn, studied outcomes may reveal any alterations to the immune response that could lead to widespread *S. aureus* infection and the role immunosuppression may play in the unusual clinical presentation observed.

## Materials and Methods

### Birds and Inoculation

All procedures involving animals were monitored and approved by the Iowa State University Institutional Animal Care and Use Committee. Hy-Line W-36 laying hens at 22- and 96-weeks of age were obtained (100/age group) from the commercial egg complex from the previous outbreaks, transported to the Iowa State University Laboratory Animal Research Center and evenly distributed between 4 rooms and housed in raised floor pens (25 birds/pen; 2 pens/room, 64 square feet or 2 sq ft usable space/hen) with equal age representation. Hens were given a 72 h acclimation period prior to the start of the study and had *ad libitum* access to water and a standard laying hen diet. On d0, 4 birds/age group were selected for baseline blood and spleen sampling before challenge with *S. aureus* isolated from affected birds. One room was designated for a negative control group and given 1 ml sterile phosphate buffered saline by oral gavage. The remaining rooms were designated for inoculation with 1.2 × 10^8^-1.5 × 10^8^ colony forming units (CFUs) per bird of *S. aureus* by 1 of 3 routes: oral gavage, subcutaneous injection into the comb, or intravenous injection into the brachial vein. This resulted in 8 total treatments arranged in a 2 × 4 factorial of age (22- or 96-weeks-old) and inoculation route (negative control, oral gavage, subcutaneous injection, or intravenous injection).

### Physiological Responses

This work represents immunological evaluations as part of a larger challenge study (2). Among other clinical signs observed, body weight and body temperature were collected as general physiological responses to *S. aureus* inoculation. Individual hen body weight and cloacal body temperatures were collected daily following inoculation.

### Seahorse Metabolic Assay

Blood was collected from the brachial vein of 4 birds/ treatment at baseline, 6 h post-inoculation (pi), 24 hpi, 3 dpi, and 7 dpi into heparin-coated collection tubes. Peripheral blood mononuclear cells (PBMC) were isolated from whole blood using Histopaque 1077 and 1119 (Sigma-Aldrich, St. Louis, MO, USA). Isolated cells were counted using a hemocytometer and plated at a density of 3 × 10^6^ cells/well for use in the Cell Energy Phenotype Test Kit (Agilent; 103325-100) within the Seahorse XFe24 Analyzer system at 40°C (Agilent, Santa Clara, CA, USA). The Cell Energy Phenotype Test measures both mitochondrial respiration through oxygen consumption rate (OCR) and glycolysis through lactic acid production/extracellular acidification rate (ECAR) before and after a metabolic pathway inhibitor challenge with simultaneous injection of trifluoromethoxy carbonylcyanide phenylhydrazone (FCCP) and oligomycin after recording 3 baseline OCR and ECAR measurements. When added to the cell culture media (Agilent base media + 1 mM pyruvate, 2 mM glutamate, and 10 mM glucose), oligomycin inhibits mitochondrial respiration and causes a shift toward glycolytic metabolism (i.e., ECAR values increase), whereas FCCP injection depolarizes the mitochondrial membrane and causes an increase in oxygen consumption (i.e., OCR values increase) (11). Simultaneous FCCP and oligomycin injection causes a compensatory shift in metabolism toward either anaerobic glycolysis or mitochondrial respiration to provide insight into cellular metabolic potential and preference. Following injection with FCCP and oligomycin, the XFe24 Analyzer takes 5 additional OCR and ECAR measurements. To calculate metabolic potential from raw ECAR and OCR values, stressed values (post-assay inhibitor challenge) were divided by baseline metabolic readings and multiplied by 100. When presented this way, values > 100% indicate an increase in ECAR/OCR over baseline when stressed, values <100% suggest a reduction compared to baseline, and values = 100% indicate no response to FCCP or oligomycin.

### Flow Cytometry

Splenocytes were isolated from 4 euthanized birds/ treatment at baseline, 3 and 8 dpi by gently homogenizing collected spleens in PBS and passing the solution through a 70 μm sterile cell strainer. PBMC were isolated as previously described. Four aliquots of collected splenocytes and PBMC not used in the Seahorse metabolic assays were frozen in chicken serum with 7.5% DMSO at −80°C until analysis.

Immune cell profiles were detected by extracellular marker flow cytometric analysis. Frozen cells were thawed, enumerated, and aliquoted into polystyrene flow cytometry tubes. PBMC from 2 birds/treatment/timepoint were pooled to obtain adequate cell numbers for analysis resulting in 2 groups of pooled PBMC samples/treatment at each timepoint. Cells in each aliquot were stained for extracellular surface markers by diluting 0.5 μl of fluorochrome-conjugated antibody in 50 μl of PBS and allowing the cells to incubate at 4°C in the dark for 30 min. The following antibodies were used to detect extracellular markers of innate immune cells and T lymphocytes: mouse anti-chicken CD1.1 FITC (clone CB3; mouse IgG_1_κ), CD3 Pacific Blue™ (clone CT-3; mouse IgG_1_κ), CD4 Alexa Fluor® 700 (clone CT-4; mouse IgG_1_κ), CD8α SPRD (clone CT-8; mouse IgG_1_κ), TCRγδ PE (clone TCR-1; mouse IgG_1_κ), and monocyte/macrophage biotin (clone KUL01; mouse IgG_1_κ; Southern Biotech, Birmingham, AL). Each tissue was stained with fluorescence-minus-one controls and associated isotype controls (0.2/50 μl PBS) were used to account for non-specific binding by each antibody. After primary staining, cells were washed in PBS and a Brilliant Violet™ (BV) 785-conjugated streptavidin secondary stain (BioLegend, San Diego, CA) was applied (0.3/50 μl PBS) to allow binding of the BV785 fluorochrome to biotin-conjugated monocyte/macrophage antibody. Cells were incubated at 4°C in the dark for 30 min, washed, and resuspended in PBS prior to analysis. Cell populations were analyzed by the BD FACSCanto™ (BD Biosciences, San Jose, CA) cytometer and individual cell populations were analyzed by FlowJo (version 10.5.0) software. Within the CD3^+^ T cell subpopulations examined (CD3^+^CD4^+^, CD3^+^CD8α^+^, and CD3^+^TCRγδ^+^), groups designated as “other” distinguish cells within the CD3^+^ cell gate that did not show staining for the selected markers.

### Statistics

Immune cell metabolic data and cellular profiles in the spleen and PBMC were analyzed using the following model:

$$\begin{array}{l} y_{ijk}=\;\mu +\;A_{i}+\;R_{j}+\;{\left(A\times \;R\right)}_{ij}+\;e_{ijk} \end{array}$$

where y_ijk_ is the observed effect (cell population), μ is the overall mean, A_i_ is the main effect of age at the ith level (*i* = 2; 22 or 96 week), R_i_ is the main effect of inoculation route at the jth level (*j* = 4; control, oral gavage, subcutaneous, or intravenous injection), (A × R)_ij_ is the interaction of bird age and inoculation route, and e_ijk_ is the random error. Analysis using this model was done using the MIXED procedure (SAS 9.4) with significance observed at *P* ≤ 0.05.

## Results

### Physiological Response to Challenge

Young (22-week) birds in the intravenous injection group showed significantly increased body temperature at 2 and 3 dpi by 1.45°F in addition to 7.5–16.8% reductions in body weight from 2–8 dpi in intravenously-inoculated hens at both ages. *Staphylococcus aureus* was isolated at 3, 5, and 8 dpi from the challenge groups but not the negative control group. The 22- week-old hens in the intravenous inoculation group showed 52% lame birds (13/25); however, none of the study groups showed the same clinical signs observed in the naturally occurring outbreaks. Additionally, no mortalities were recorded in any of the treatment groups over the course of the study (2).

### Metabolic Phenotypes

**Figure 1:**
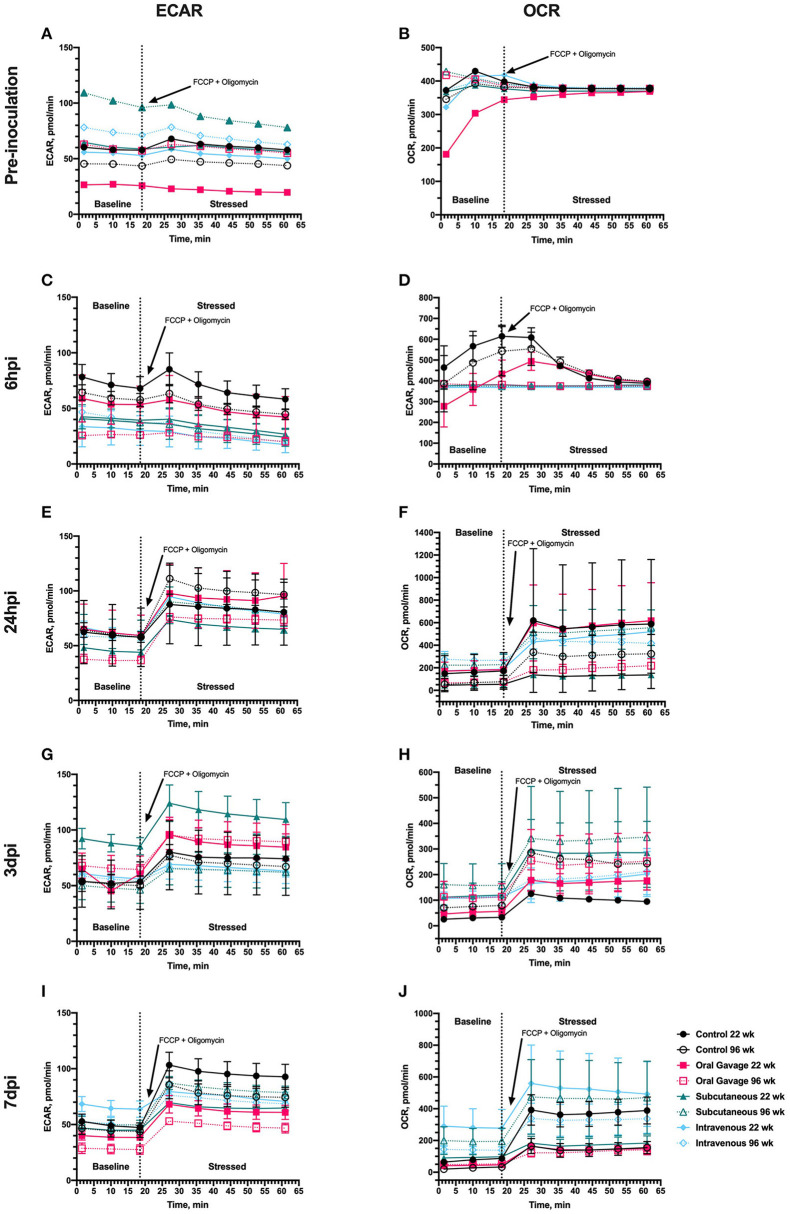
Metabolic outputs for extracellular acidification rate (ECAR) and oxygen consumption rate (OCR) of peripheral blood mononuclear cells in 22- and 96-week-old laying hens inoculated with *Staphylococcus aureus* by various routes. Outputs were collected during the Cell Energy Phenotype Test conducted **(A,B)** prior to inoculation and **(C,D)** 6 h, **(E,F)** 24 h, **(G,H)** 3 d, and **(I,J)** 7 d post-inoculation provided by the Seahorse XFe24 analyzer (Agilent, Santa Clara, CA). The dashed line separates measurements taken before (baseline) and after cells were stressed with simultaneous injection of FCCP and oligomycin to induce compensation by glycolytic (ECAR) or oxidative (OCR) pathways. Each data point represents the mean measurement taken from 3 hens/treatment ± SEM.

The Seahorse metabolic phenotype test provides several pieces of information about cellular energy selection outcomes. The baseline phenotype is a measure of the cells' relative use of mitochondrial respiration (OCR) and glycolysis (ECAR) under unaltered starting conditions, the stressed phenotype measures the relative mitochondrial respiration and glycolysis when the cell is forced to using a drug cocktail, and metabolic potential is the calculated percent change or measurement of ability to respond to the cocktail. When raw data are presented by treatment over baseline and time post-inoculation, PBMC populations did not respond to FCCP or oligomycin in the expected manner until 24 h post-inoculation, suggesting that additional stressors other than *S. aureus* inoculation alter metabolic phenotypes in these cells (Figure 1). Baseline ECAR and OCR measures at the pre-inoculation timepoint (Figures 1A,B) are non-uniform, and in the absence of an inoculation challenge, is inferencing other stressors altered metabolism. At 6 hpi, there was a significant main effect of route where subcutaneous and intravenous routes maintain a non-responsive OCR phenotype following FCCP and oligomycin administration (*P* = 0.01).

**Figure 2:**
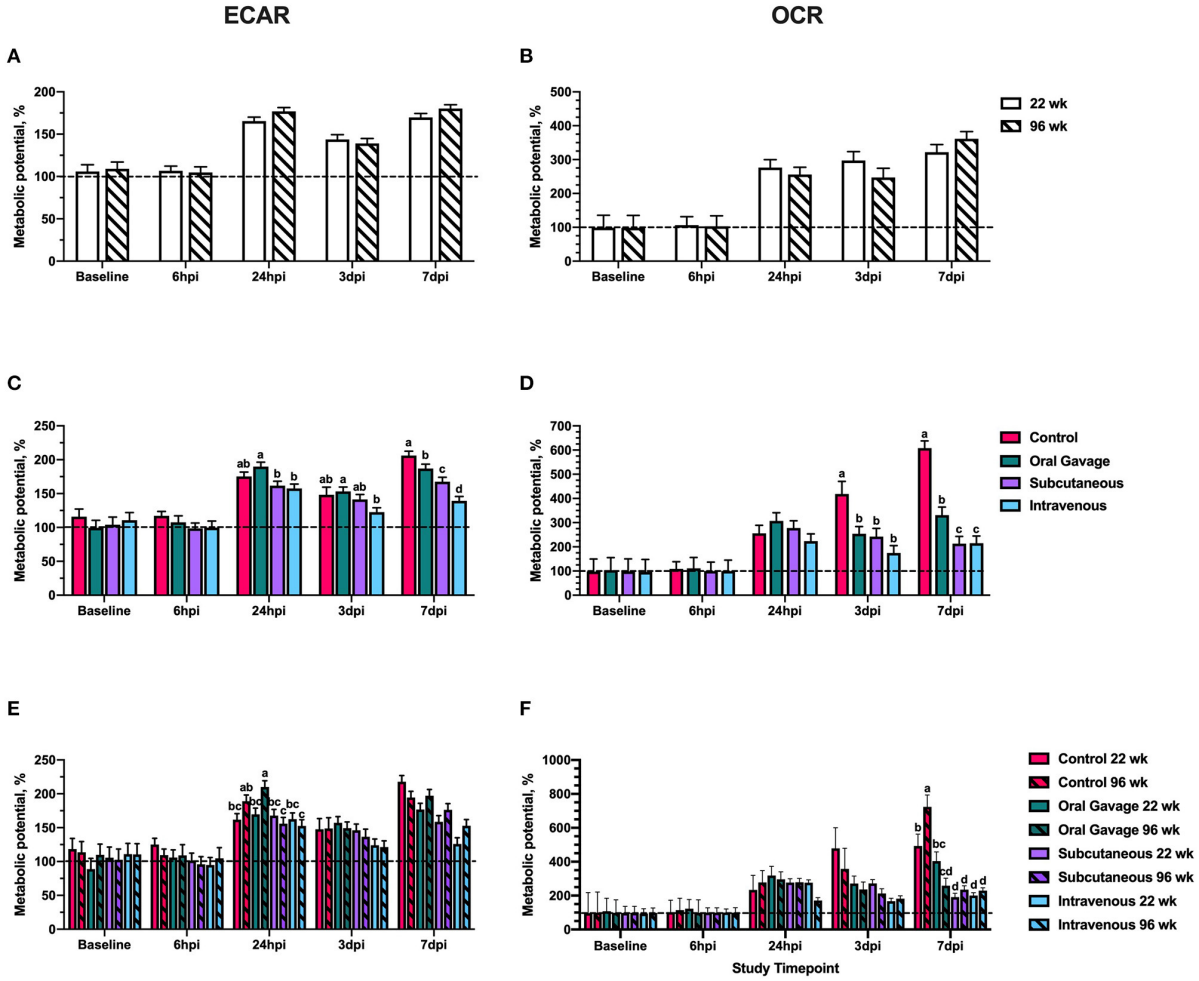
Extracellular acidification rate (ECAR) and oxygen consumption rate (OCR) metabolic potential of peripheral blood mononuclear cells in 22- and 96-week-old hens inoculated with *Staphylococcus aureus* by various routes. **(A,B)** represent the main effect of age **(C,D)** represent the main effect of inoculation route, and **(E,F)** represent the age × inoculation route interaction for ECAR and OCR, respectively. Data represent the mean metabolic potential calculated for each measurement as ((stressed/baseline) × 100) ± SEM. The dashed line at 100% represents the value where the stressed measurement = baseline measurement. Bars with different letter superscripts are statistically significant (*P* ≤ 0.05).

When expressed as a metabolic change in response to oligomycin and FCCP, no differences in ECAR or OCR metabolic potential were observed at baseline and 6 hpi (Figure 2). The metabolic potential was ~100% at both timepoints, suggesting an overall non-response to FCCP or oligomycin (Figure 2) that further corroborates with outcomes reported in Figure 1. When timepoint is included in the statistical model, ECAR and OCR metabolic potential is significantly increased in all ages and inoculation routes starting at 24 hpi (*P* < 0.0001), indicating metabolic alterations in response to inoculation challenge. Differences between baseline and stressed OCR and ECAR measurements observed at 24 hpi also support a return to responsiveness between 24 h and 3 dpi for ECAR and OCR.

ECAR metabolic potential between *S. aureus*-inoculated groups did not differ from control until 7 dpi, when all inoculation routes showed significant reduction in the ability of PBMC to utilize anaerobic (glycolytic) metabolism to meet energy demands. At this timepoint, intravenously inoculated birds had the greatest reduction in ECAR metabolic potential compared to the control (32.4%) whereas hens orally inoculated with *S. aureus* showed only a 9.3% reduction (*P* = 0.0003; Figure 2C). As increased glycolytic activity is associated with an increased inflammatory response, these outcomes would suggest that *S. aureus* could be inhibiting the immune response by altering the immune cell ability to metabolically activate, particularly when pathogens are introduced by subcutaneous or intravenous injection. In contrast to ECAR, changes to OCR metabolic potential were detected at 3 dpi when all inoculated groups showed 39.3–48.2% reductions in metabolic potential compared to the control (*P* = 0.001). Similarly, all inoculated groups at 7 dpi had a 45.6–48.2% reduction in the ability to use oxidative metabolism to meet metabolic demands, with orally-inoculated birds having less severe reductions (45.6%) compared to subcutaneous and intravenous injection groups (65.0%; *P* < 0.0001; Figure 2D). Such outcomes indicate that oxidative metabolism is universally reduced by *S. aureus* in the later stages of inoculation, but the inoculation route may determine the impact on glycolytic metabolism (inflammatory response).

### Systemic Immune Cell Populations

Baseline measurements in healthy laying hens showed that 96-week-old birds had 26.7 and 12.3% fewer CD3^+^ T cells than their younger counterparts in both the PBMC and spleen, respectively (*P* = 0.03 and 0.0003). When examining underlying T cell populations, 96-week-old birds had consistently higher CD3^+^CD4^+^ helper T cell populations (T_H_; 16.7 and 28.9%) and CD3^+^CD8α^+^ cytotoxic T cells (T_C_; 16.8 and 7.7%) in both the PBMC and spleen, respectively, with significant reductions in CD3^+^TCRγδ^+^ (γδ) T cells by 60.5 and 36.3% (*P* < 0.05; Supplementary Figure 1). Other measured immune cell populations in the PBMC comprised a small percentage of the total live cells (<10%), with PBMCs generally having fewer monocyte/macrophage^+^ cells and more CD1.1^+^ antigen presenting cells (APCs) than the spleen.

**Figure 3:**
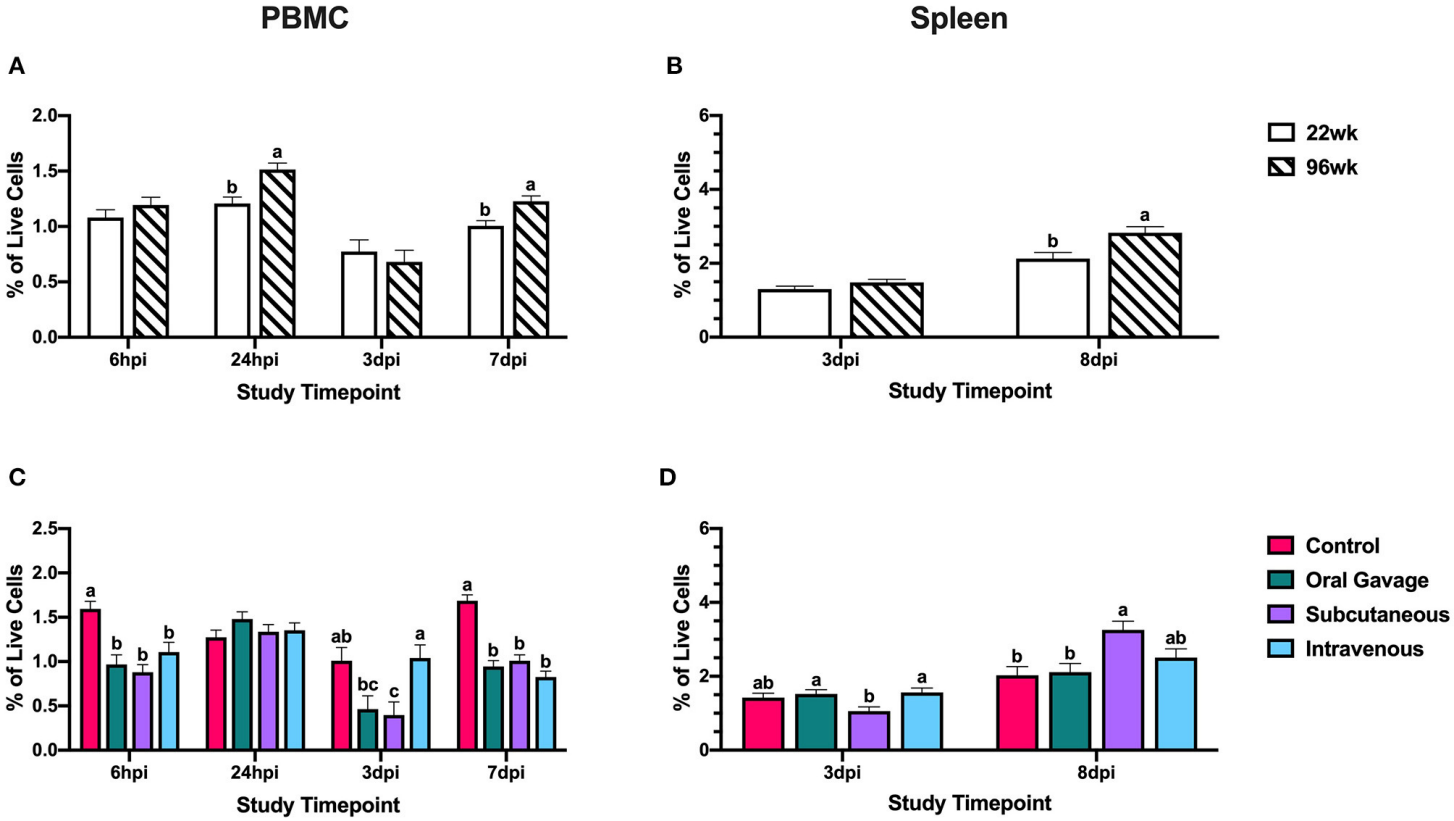
Systemic monocyte/macrophage^+^ cells in 22- and 96-week-old hens inoculated with *Staphylococcus aureus* by various routes. **(A,B)** represent the main effect of hen age and **(C,D)** represent the main effect of inoculation route for the peripheral blood mononuclear cells (PBMC) and spleen, respectively. Data represent the mean ± SEM of all live cells with the monocyte/macrophage marker detected by flow cytometry. Bars with different superscripts within each tissue and timepoint are significantly different (*P* ≤ 0.05).

At 3 dpi, monocyte/macrophage^+^ cells in PBMC were reduced 60.7% compared to the control in subcutaneously inoculated groups (*P* = 0.02); however, these populations in the spleen did not differ from the control at the same time. This would indicate that circulating monocyte/macrophage^+^ cells were not recruited to the spleen at 3 dpi but may be moving directly to the inoculation site in subcutaneously inoculated hens. At 7 dpi, monocyte/macrophage populations in PBMC were reduced 40.2–51.0% compared to control in all inoculated groups, but only subcutaneously-inoculated hens showed an increased splenic population by 37.8% compared to the negative control around the same time (8 dpi; *P* < 0.0001; Figure 3). This suggests that monocyte/macrophage^+^ cells in subcutaneously-inoculated birds were being recruited to the spleen for potential antigen presentation and initiation of an adaptive immune response in the last stages of the inoculation.

**Figure 4:**
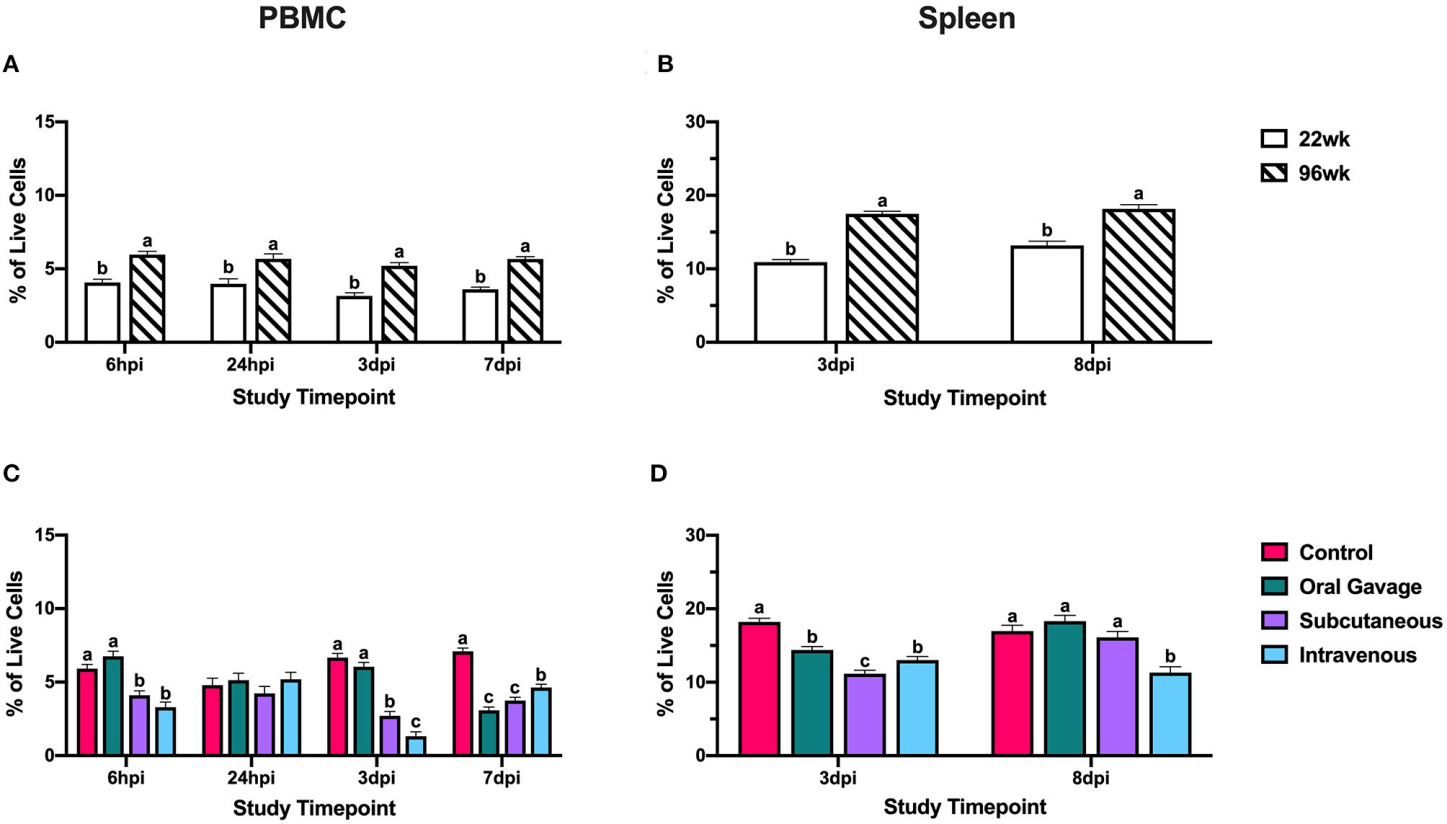
Systemic CD1.1^+^ cells in 22- and 96-week-old hens inoculated with *Staphylococcus aureus* by various routes. **(A,B)** represent the main effect of hen age and **(C,D)** represent the main effect of inoculation route for the peripheral blood mononuclear cells (PBMC) and spleen, respectively. Data represent the mean ± SEM of all live cells with the CD1.1 marker detected by flow cytometry. Bars with different superscripts within each tissue and timepoint are significantly different (*P* ≤ 0.05).

Ninety-six-week-old hens consistently had significantly higher CD1.1^+^ cells than their younger counterparts in the spleen (27.4–37.6%) and PBMC (29.9–39.4%; *P* < 0.05; Figures 4A,B). These cell populations remained fairly stable throughout the post-inoculation period between the two age groups, indicating that bird age does not significantly contribute to CD1.1^+^ cell responses to *S. aureus*. Hens inoculated by subcutaneous or intravenous injection had significantly fewer CD1.1^+^ cells than the control by 59.3 and 80.1% at 3 dpi with simultaneous reductions in the spleen by 21.1–38.3% (*P* < 0.0001). While orally inoculated birds also showed reductions in splenic CD1.1^+^ cells at 3 dpi, the lack of a response by these cells in PBMC suggests that cells may have been recruited to the inoculation site directly from the spleen without a systemic reduction in CD1.1^+^ cells. At the conclusion of the study (7 and 8 dpi), the PBMC of all birds inoculated with *S. aureus* had 34.8–56.5% fewer CD1.1^+^ cells than the control whereas only splenic CD1.1^+^ cells were reduced by 33.3% in intravenously inoculated hens (*P* < 0.0001; Figures 4C,D). CD1.1^+^ APCs in orally and subcutaneously inoculated birds may have been recruited to the spleen for the initiation of an adaptive response at 7 dpi, but global reductions in this cell type were still occurring at later timepoints in intravenously inoculated hens.

**Figure 5:**
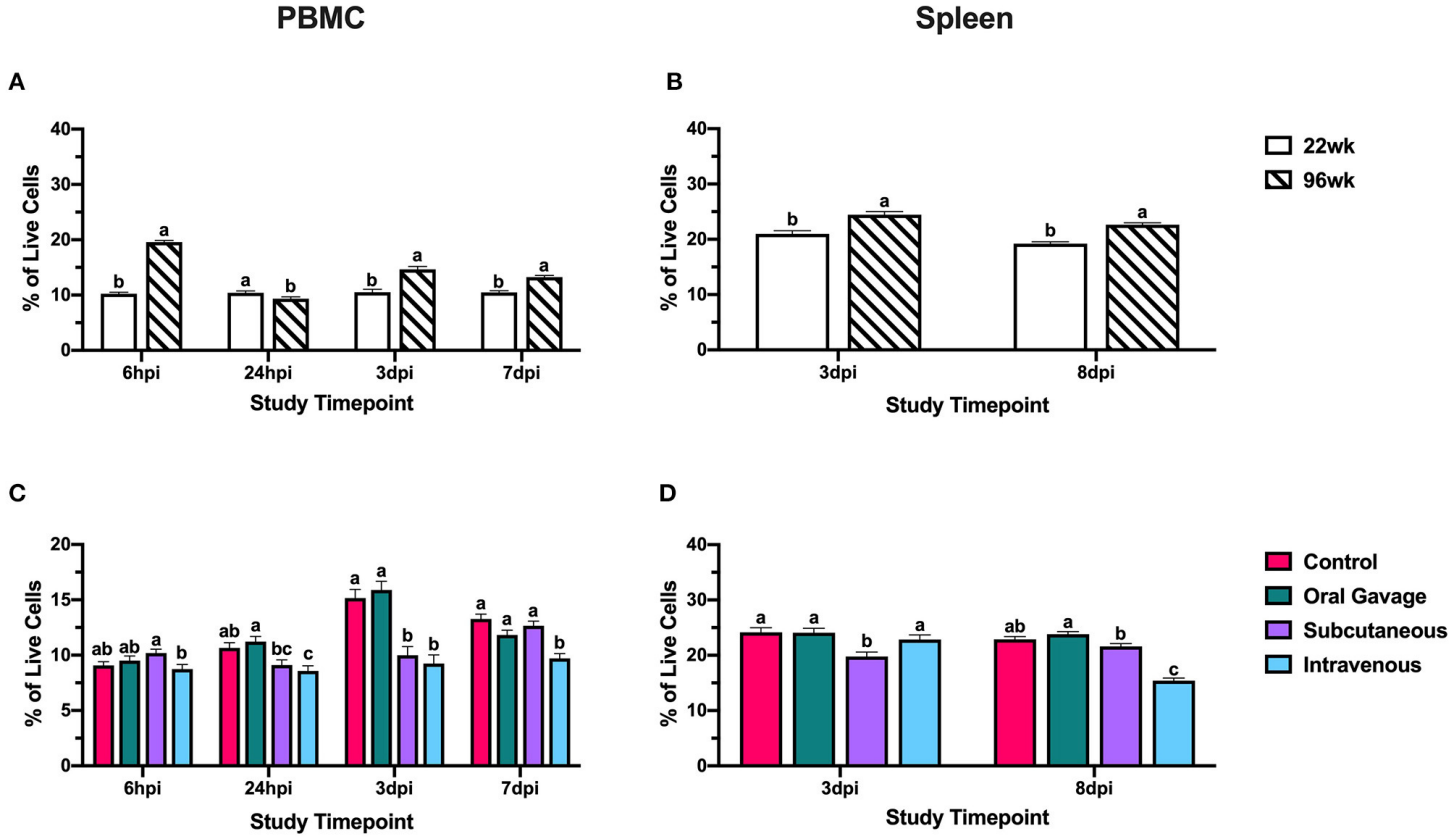
Systemic CD3^+^ T cells in 22- and 96-week-old hens inoculated with *Staphylococcus aureus* by various routes. **(A,B)** represent the main effect of hen age and **(C,D)** represent the main effect of inoculation route for the peripheral blood mononuclear cells (PBMC) and spleen, respectively. Data represent the mean ± SEM of all live cells with the CD3 marker detected by flow cytometry. Bars with different superscripts within each tissue and timepoint are significantly different (*P* ≤ 0.05).

Throughout the post-inoculation period, 96-week-old birds generally had 21.0–47.7% increased percentages of CD3^+^ cells in the PBMC and 14.2–15.2% enriched in the spleen (*P* < 0.0001; Figures 5A,B). Notably, these PBMC populations in 96-week-old birds showed fluctuation across the different timepoints, whereas 22-week-old hens retained more stable populations throughout the trial (Figure 5A). Control and oral gavage groups showed fluctuating and responsive T cell populations in the PBMCs over the course of the challenge period while populations in subcutaneous and intravenous groups were consistently depressed. At 3 dpi, both subcutaneous and intravenous injection groups had 34.1 and 39.0% fewer T cells, respectively, in the PBMC compared to control; however, only birds inoculated by subcutaneous injection showed an 18.1% reduction in the spleen indicating a more systemic reduction in this inoculation route (*P* < 0.0001 and = 0.007). In the final timepoints, 7 and 8 dpi, hens inoculated with *S. aureus* by the intravenous route had 26.9 and 32.7% fewer T cells in both the PBMC and spleen, respectively, compared to the control (*P* < 0.0001; Figures 5C,D). At this time, subcutaneously inoculated hens showed T cell population recovery in the PBMC and spleen to levels similar to the control, suggesting that global T cell reductions at 3 dpi were salvaged in this group while intravenously inoculated birds showed further decline.

**Figure 6:**
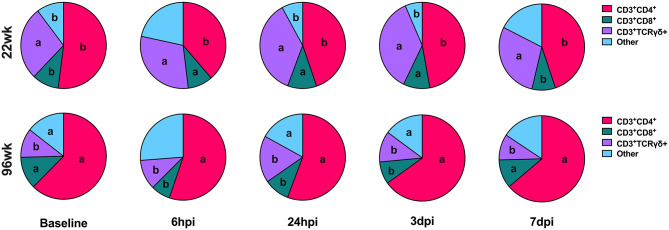
T cell subpopulations in the PBMC of 22- and 96-week-old birds inoculated with *Staphylococcus aureus*. Different letters between same-colored slices within the same timepoint are significantly different *P* ≤ 0.05.

When examining the underlying T cell populations, the general trend was that spleen populations remained relatively static over the course of the study despite noted differences in the presence of each cell type between the age groups and inoculation routes (Supplementary Figures 2, 3). Analyzed subpopulations included T_H_, T_C_, and γδ T cells with remaining CD3^+^ populations not expressing any of these markers classified as “other” T cells. At 6 hpi, both age groups showed T cell expansion within the “other” category in PBMC, displacing T_C_ cells in 96-week-old hens to populations 22.2% lower than those observed in 22-week-old birds (*P* = 0.009). In contrast, early expansion of “other” T cells in 22-week-old birds displaced T_H_ cells to levels 30.0% lower than 96-week-old birds (*P* < 0.0001). Most notably, 96-week-old birds had significantly reduced γδ T cell populations that peaked around 24 hpi but still remained 51.5% lower than their younger counterparts at the same time (*P* < 0.0001). The dominant T cell subpopulation in 96-week-old birds was T_H_ cells, which remained at levels 19.8-29.8% greater than those seen in 22-week-old birds throughout the post-inoculation period (*P* < 0.0001). At 24 hpi and 3 dpi, 96-week-old birds had 53.2 and 56.4% greater populations of “other” T cell subpopulations (*P* < 0.0001) which continued to displace T_C_ cells to levels 12.8 and 16.3% lower than those seen in their younger counterparts (*P* < 0.0001 and = 0.01; Figure 6).

**Figure 7:**
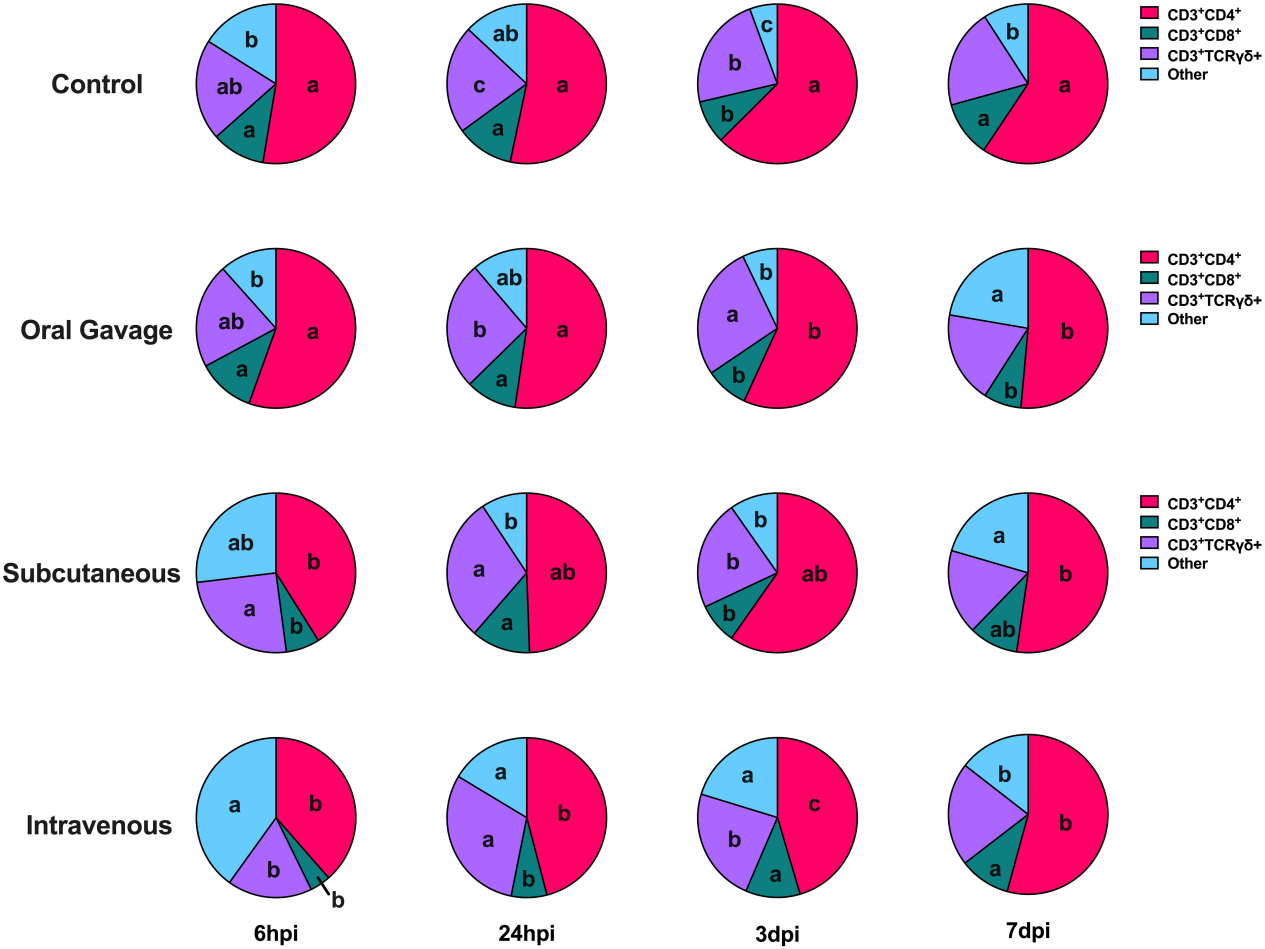
T cell subpopulations in the PBMC of birds inoculated with *Staphylococcus aureus* by various routes. Different letters between same-colored slices within the same timepoint are significantly different *P* ≤ 0.05.

Throughout the course of the study, birds in the control and oral gavage groups showed relatively static T cell subpopulations in the PBMC, while greater responsiveness was observed in the subcutaneous and intravenous injection groups. At 6 hpi, hens in the intravenous group had predominant populations of “other” T cells at levels 59.9% above control combined with 26.7% fewer T_H_ cells and 61.5% fewer T_C_ cells than the control, (*P* < 0.0001 and = 0.005). Responses in the subcutaneous injection group were characterized by continued expansion of T_H_ cells peaking at 3 dpi, resulting in displacement of T_C_, γδ T, and “other” T cells. In contrast, changes to T cell subpopulations in intravenously-inoculated hens started with early expansion of T_H_, T_C_, and γδ T cells between 6 and 24 hpi to displace cells in the predominant “other” category. At 3 dpi, T_C_ cells in intravenously inoculated hens showed continued expansion to levels 20.3% greater than control while T_H_ and γδ T cells showed minimal changes between 24 hpi and 3 dpi (*P* = 0.01). With the exception of 7 dpi, populations of “other” T cells remained significantly higher in the intravenous inoculation group throughout the study compared to the other groups, despite notable displacement over time (*P* = 0.05 and < 0.0001; Figure 7).

## Discussion

During the cell phenotype assay, the simultaneous injection of FCCP and oligomycin was expected to increase both ECAR and OCR with the resulting compensatory response providing three types of information: (1) Can the cells respond with changes to oxygen use and pH when forced to use these energy pathways; (2) If they can respond, what is the magnitude?; and (3) Did experimental variables alter this response? This step-wise process gives insight into metabolic pathways used preferentially by cultured cells when stressed (glycolysis vs. oxidative respiration). A stressor can be applied at several levels. For example, in this experiment, the birds themselves were stressed with inoculation route, and the metabolic assay added additional cellular stress *via* FCCP and oligomycin drug cocktail. From consistently altered baseline (pre-inoculation, metabolic baseline before drug administration), it is apparent that additional stressors outside of experimental variables altered outcomes early in the experiment. We can likely attribute this to acclimation stress altering immune function, as the expected response to FCCP and oligomycin was not reported in Figures 1A–C. In Figure 1D, it is apparent that inoculation route had a main effect on responsiveness in mitochondrial energy usage. Control birds indicated outside stressors altered normal response to these drugs, while outcomes observed in subcutaneous and intravenous routes suggest that these birds were experiencing additional metabolic shifts consistent with early immune responses. By 24 hpi, or ~1 week past hen arrival, the non-inoculated control hen PBMC show the expected response to FCCP and oligomycin, while inoculated hens vary based on route. By 7 dpi, oral gavage is significantly lower than all treatments for raw ECAR values (Figure 1I). Based on flow cytometry results, it was expected that orally-inoculated hens had a less robust immune response than intravenous and subcutaneous routes, but more work needs to be completed to understand this depressed anaerobic metabolic response. When considering mitochondrial respiration (Figure 1J), intravenous and subcutaneous routes showed increased oxygen consumption rates prior to drug introduction, which may be consistent with immune cell recruitment and expansion.

Considering the second outcome, or magnitude of response to a stressor, in the early timepoints of the study (baseline and 6 hpi), metabolic potentials were ~100% for all ages and inoculation routes. This additionally indicates that the cultured primary cells were non-responsive to the injected drugs and were stressed prior to the administration of FCCP and oligomycin (Figure 2). This may be explained by a number of known stressful events occurring just prior to the study, including transportation, social disruption, a new caging system and setting, and daily handling. In birds, repeated handling, conventional cage housing systems, and transportation periods as short as 30 min have been shown to increase corticosterone and suppress the immune system, while prolonged social disruption altered corticosterone to levels consistent with chronic stress in laying hens (12–16). In turn, corticosterone is associated with immunosuppressive effects such as reducing white blood cell counts, altered leukocyte trafficking, and reducing antibody titer during vaccine challenge (17, 18). Though other studies have shown no effect of transportation stress on the glycolytic potential in the breast muscle of broiler chickens, the specific effects of acute stress on the metabolic potential of laying hens is poorly described (19, 20). The baseline and 6 hpi results in this study suggest that acute stress inhibited the responsiveness of PBMC to specific metabolic inhibitors used by the Seahorse assay and an acclimation period more than 72 h is justified. In spite of this apparent immunosuppression, inoculating these birds did not result in the unusual clinical signs observed in the naturally-occurring outbreaks. This likely means that higher levels of immunosuppression combined with other pre-disposing and/or concurrent factors are involved in the naturally occurring outbreaks.

A number of immune cell population differences indicated by flow cytometry were noted at baseline and 6 hpi (Figures 5–7 and Supplementary Figure 1); however, determination of their role in immunometabolism is complicated due to a clear separate stressor that altered aerobic and anaerobic metabolic planes until 24 or more hours pi (Figures 1, 2). Hens were allowed a 72-h acclimation period before baseline sampling and experimental inoculation, but a clear lack of cellular responsiveness to experimental drugs that force use of metabolic pathways in all hens, including controls, indicates stressors other than age or route of inoculation. For experiments of this type, it is possible that 5 or more days would improve metabolic experimental outcomes in the early phases. Transport stress, change in housing conditions from conventional cages to floors, and the general effects of corticosterone have been associated with increased circulating innate immune cells (particularly heterophils), decreased lymphocytes, and increased populations of T_H_ and T_C_ cells (17, 18, 21). No observable reductions in monocyte/macrophage or CD1.1^+^ populations were observed between 6 and 24 hpi, which roughly corresponds with the reduction of background stress in metabolic assays. This would indicate that these cell populations were not likely elevated by stress; however, heterophils are more commonly associated with altered innate immunity during stress and were not analyzed in this study due to a lack of available immunological reagents. Baseline and 6 hpi populations of CD3^+^ T and T_H_ cells in 96-week-old hens reported here were higher than those observed in the PBMC of healthy laying hens by other reports, while populations in 22-week-old birds were more similar (22, 23). It is important to note that lymphocyte responses to stress are time-dependent (18). These observations combined with high body weight losses in 96-week-old birds during the acclimation period reported by Meyer et al. suggest that 22-week-old birds may have faster lymphocyte recovery contributing to less severe body weight loss following stress compared to their older counterparts but does not translate to a return in metabolic potential (2).

When metabolic potentials showed a recovered responsiveness to FCCP and oligomycin at 24 hpi, OCR potentials were typically greater than ECAR potentials (>200 vs. <200%), suggesting preferential use of oxidative pathways by PBMC to meet metabolic demands during stress (Figure 2). Notably, bird age did not affect PBMC capability to respond to metabolic stress; however, the general trend was a decreased ability to increase either oxidative or glycolytic metabolism to meet metabolic needs in *S. aureus* inoculated birds compared to the control. Birds inoculated by oral gavage showed the lowest magnitude of change in metabolic potential while intravenously inoculated birds were numerically and significantly lower than other inoculation routes over the course of the study. Differences between oral and intravenous routes are expected as physiological barriers like ventriculus pH and competitive exclusion by the intestinal microbiota may reduce the ability of orally introduced pathogens to colonize, whereas fewer physiological barriers exist when bacteria are directly introduced to the blood (24–26).

Two populations of APCs were measured in this study, monocyte/macrophages and CD1.1^+^ cells. Between the two different lineages, monocyte/macrophage^+^ cells play a dual role in pathogen clearance during the innate response and antigen presentation to initiate an adaptive response, while CD1.1^+^ cells are classically associated with lipid-antigen presentation (27, 28). Low populations of monocyte/macrophages in PBMC were expected as macrophages are found primarily in peripheral tissues and their monocyte precursors are found at low percentages in avian blood (29, 30). Reductions in monocyte/macrophage^+^ cells at 3 dpi in orally and subcutaneously-inoculated birds without splenic involvement suggested recruitment to inoculation sites for pathogen clearance in the innate immune response. Notably, intravenously inoculated birds do not show the same reduction, and populations within the blood stay relatively stable over time; however, it is important to note that examination of these cells in PBMC encompasses examination at the inoculation site in this group. In the late stages of the inoculation, only subcutaneously inoculated birds show increased monocyte/macrophage^+^ cells in the spleen suggesting that these cells may be presenting antigen to splenic T cells to initiate the adaptive response. In contrast, orally inoculated birds show no difference in splenic populations to indicate antigen presentation while intravenously inoculated birds show a small numerical increase that may suggest some initiation of an adaptive response that is less robust than that achieved by birds in the subcutaneously inoculated group (Figure 3). This observation may suggest that local insults (e.g., skin scratches, injuries, etc.) may be the likely route of *S. aureus* introduction inducing the observed clinical lesions and the systemic effects are secondary to this event.

Within CD1.1^+^ cells, subcutaneous and intravenous injection groups showed systemic losses to CD1.1^+^ cells at 3 dpi, while only splenic populations were reduced in the orally gavaged group. This further suggests that oral inoculation results in less severe immune impairment compared to other inoculation routes. While CD1.1^+^ cells were reduced in the PBMC across all treatments at 7 dpi, subcutaneously inoculated hens showed splenic recovery of these cells while intravenous groups still had significantly reduced CD1.1^+^ cells in both tissues (Figure 4). While the specific role of lipid antigen presentation during *S. aureus* infection is unclear, this reduction in APCs at later timepoints suggests some impairment in the ability to initiate the adaptive immune response in intravenously inoculated hens.

When naïve T cells encounter antigen, metabolic phenotypes transition from oxidative respiration to anaerobic glycolysis, contributing to increased ECAR measurements which can be interpreted as an inflammatory response (31). Increased T cell activation would be typically expected in the later post-inoculation timepoints (7 dpi) as antigen is presented to these cells by the innate immune system and the adaptive response initiates. Notably, all inoculated birds showed reductions in the ability to utilize anaerobic glycolysis to meet metabolic demands at 7 dpi which is suggestive of impaired T cell activation. This may be partly due to the low inflammatory response and clinical presentation (2) but may also be explained by differential T cell populations. At 7 dpi, T_H_ cells were significantly reduced in the PBMC of all inoculated birds (Supplementary Figure 4). As these T cell subpopulations are critical for the activation of T_C_ cells and other effector lymphocytes, simultaneous reductions in this cell type and ECAR metabolic potential supports metabolic observations that *S. aureus* impairs T cell responses in laying hens (32).

As previously mentioned, intravenously inoculated birds showed the greatest metabolic inhibition during the post-inoculation timepoints, which may be explained by further discrepancies observed between inoculation groups. Hens in the intravenous group had significantly reduced overall CD3^+^ T cell presence in both the PBMC and spleen compared to the other inoculation groups (Figure 5). Within the T cell subpopulations, the intravenous inoculation group also had the greatest population of “other” cells in the early days of the inoculation at the expense of other T cell subpopulations. As a result, comparatively greater changes in T_H_, T_C_, and γδ occurred over the course of the post-inoculation period to compensate for early disparities (Figure 7). At the conclusion of the study, T cell subpopulations in the intravenous group were more similar to those observed in the other inoculation groups; however, the enhanced early expansion of these subpopulations combined with significantly reduced overall T cell presence may have had an additive effect on metabolic impairment in this group.

**Figure 8:**
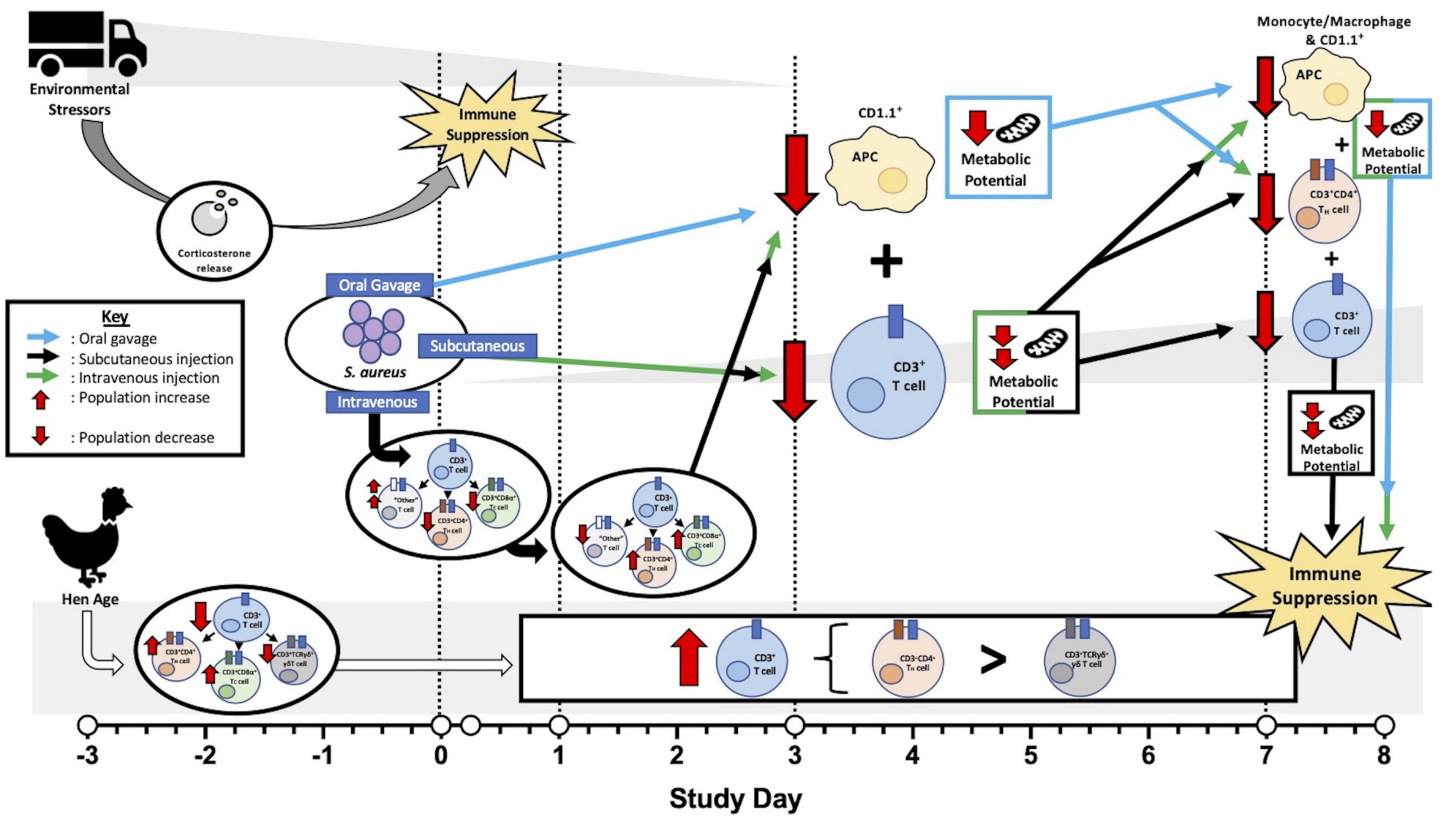
Overview of factors contributing to laying hen immunity before and after *Staphylococcus aureus* inoculation. Prior to inoculation, environmental stressors related to transport, handling, and social disruption suppressed peripheral blood mononuclear cell (PBMC) metabolic potential in all hens with resolution occurring at later timepoints. Baseline T cell populations were reduced in older (96-week) laying hens with subpopulations favoring T helper (T_H_) and cytotoxic (T_C_) cell populations over γδ T cells. Throughout the post-inoculation period, older hens maintained higher T cell populations than their younger (22-week) counterparts with subpopulations favoring T_H_ subtypes over potentially regulatory γδ T cells. Generally, hens inoculated by oral gavage experienced mildly reduced metabolic potential while subcutaneous and intravenous injection groups had intermediate and severe reductions, respectively. Losses in both antigen presenting cells (APC; 3 and 7 dpi) and T_H_ cells (7 dpi) in all *S. aureus*- inoculated hens may be responsible for universal metabolic potential reduction. Simultaneous T cell reduction at 3 dpi in both the subcutaneous and intravenous injection groups may have an additive effect on metabolic impairment with T cell recovery in subcutaneous groups contributing to intermediate metabolic effects. Shifts in T cell subpopulations in the first 24 hpi and continued T cell losses at 7 dpi in intravenously-inoculated hens potentially contributed to severely reduced metabolic potential. As metabolic potential can be linked to immune function, these outcomes support immune suppression as a result of these various fact.

While age did not impact metabolic potential in this study, notable differences in T cells and their subpopulations in 96-week-old birds were observed. These hens had significantly reduced CD3^+^ T cells at baseline that increased early (6 hpi) in both the spleen and PBMC and generally remained higher than their younger counterparts for the remainder of the study (Figure 5). Within these T cells, 96-week-old birds consistently had higher populations of T_H_ cells than their younger counterparts and significantly reduced γδ T cells, regardless of post-inoculation timepoint (Figure 6). The function of γδ T cells in poultry is not well-defined, but they are linked to responses to bacterial infection and generally regarded as playing a regulatory role in the immune response (33). The reduced presence of γδ T cells in 96-week-old laying hens combined with increased T_H_ presence and minimal febrile response reported by Meyer et al. suggests that older hens may have a less responsive and regulated immune response possibly due to prolonged time in a commercial environment or previous exposure to *S. aureus* (2). Ultimately, these factors may contribute to varying clinical presentation during experimental *S. aureus* infection between young and old hens. An overview of specific immune and metabolic observations linked to hen age, environmental stressors, and *S. aureus* inoculation route is detailed in Figure 8.

Overall, the results provide insights into the immunosuppressive effects of environmental and transport stress that may change the length of acclimation periods used by similar challenge studies in the future. While early metabolic and immune responses to *S. aureus* were obscured by environmental or transport stress in the early study timepoints, later reductions in T_H_ cells across all treatment groups combined with reductions in glycolytic potential suggest that *S. aureus* initially inhibits T cell metabolic activation and the downstream adaptive immune response. Despite the observed stress early in this study, challenging birds with *S. aureus* alone was not sufficient to recreate the disease noted in commercial production facilities. This suggests that stronger stressors, other primary pathogens, or a combination of several stressors could have pre-disposed the naturally occurring outbreaks. Observed differences between inoculation routes suggest an additive effect of immune profiles on metabolic responses. Intravenously inoculated birds demonstrated impaired antigen presentation contributing to delayed adaptive responses combined with generally lower T cell populations and greater expansion requirements within T cell subpopulations. These factors may have all contributed to the generally greater impairments to metabolic potential observed in these hens, suggesting a connection between T cell fluctuations and metabolism that requires additional research. Aged hens with reduced γδ T cell populations may be more susceptible and slower to respond to the collective effect of pathogenic infections as compared to younger hens. While it is unlikely that *S. aureus* was the primary pathogen in the unusual hen mortality event that was the premise for this work, the work presented here suggests that hen age, transport stress, and inoculation route are factors to consider when assessing systemic infection and high mortality in laying hens.

## Data Availability Statement

The raw data supporting the conclusions of this article will be made available by the authors, without undue reservation.

## Ethics Statement

The animal study was reviewed and approved by Iowa State University Institutional Animal Care and Use Committee.

## Author Contributions

YS, ME-G, and EB contributed to the conceptualization and design of the study. MM, YS, ME-G, and EB performed live animal experiments. KF-C and MM performed laboratory experiments and associated data analysis. KF-C wrote the manuscript. All authors contributed to interpretation of the results and manuscript editing.

## Conflict of Interest

The authors declare that the research was conducted in the absence of any commercial or financial relationships that could be construed as a potential conflict of interest.

## Abbreviations

CFU: colony forming unit
pi: post-inoculation
PBMC: peripheral blood mononuclear cells
FCCP: trifluoromethoxy carbonylcyanide phenylhydrazone
OCR: oxygen consumption rate
ECAR: extracellular acidification rate
T_H_: helper T cell
T_C_: cytotoxic T cell.
