# Supplementary material for: Age and Staphylococcus aureus Inoculation Route Differentially Alter Metabolic Potential and Immune Cell Populations in Laying Hens
Source: Front Vet Sci. 2021 Mar 26;8:653129. doi: 10.3389/fvets.2021.653129 (PMC8032939; doi:10.3389/fvets.2021.653129)
Supplement: Supplementary file 1 [file Data_Sheet_1.docx]

***Supplementary Material***

.

**Supplemental Figure 1:** Immune cells measured in the peripheral blood mononuclear cells (PBMC) and spleen of healthy 22- and 96-week-old hens prior to inoculation with *Staphylococcus aureus* by various routes. Panels **(A)** and **(B)** represent antigen presenting cells while panels **(C)** and **(D)** represent the overall T cells and underlying subpopulations within the peripheral blood mononuclear cells (PBMC) and spleen, respectively. Data represent the mean ± SEM of all live cells or cells within either the CD1.1^+^ gate (CD1.1^+^CD8α^+^) or CD3^+^ gate (CD3^+^CD4^+^, CD3^+^CD8α^+^, CD3^+^TCRγδ^+^) detected by flow cytometry. Bars with different superscripts within each tissue and timepoint are significantly different (*P* ≤ 0.05).

**Supplemental Figure 2**: T cell subpopulations in the spleens of 22- and 96-week-old birds inoculated with *Staphylococcus aureus*. Different letters between same-colored slices within the same timepoint are significantly different *P* ≤ 0.05.

**Supplemental Figure 3**: T cell subpopulations in the spleens of birds inoculated with *Staphylococcus aureus* by various routes. Different letters between same-colored slices within the same timepoint are significantly different *P* ≤ 0.05.

**Supplemental Figure 4:** Systemic CD3^+^CD4^+^ helper T cells in 22- and 96-week-old hens inoculated with *Staphylococcus aureus* by various routes. Panels **(A)** and **(B)** represent the main effect of hen age and **(C)** and **(D)** represent the main effect of inoculation route for the peripheral blood mononuclear cells (PBMC) and spleen, respectively. Data represent the mean ± SEM of all CD4^+^cells within CD3^+^ populations detected by flow cytometry. Bars with different superscripts within each tissue and timepoint are significantly different (*P* ≤ 0.05).

**Supplemental Figure 5:** Systemic CD3^+^CD8α^+^ cytotoxic T cells in 22- and 96-week-old hens inoculated with *Staphylococcus aureus* by various routes. Panels **(A)** and **(B)** represent the main effect of hen age and **(C)** and **(D)** represent the main effect of inoculation route for the peripheral blood mononuclear cells (PBMC) and spleen, respectively. Data represent the mean ± SEM of all CD8α^+^ cells within CD3^+^ populations detected by flow cytometry. Bars with different superscripts within each tissue and timepoint are significantly different (*P* ≤ 0.05).

**Supplemental Figure 6:** Systemic CD3^+^TCRγδ^+^ T cells in 22- and 96-week-old hens inoculated with *Staphylococcus aureus* by various routes. Panels **(A)** and **(B)** represent the main effect of hen age and **(C)** and **(D)** represent the main effect of inoculation route for the peripheral blood mononuclear cells (PBMC) and spleen, respectively. Data represent the mean ± SEM of all TCRγδ^+^ cells within CD3^+^ populations detected by flow cytometry. Bars with different superscripts within each tissue and timepoint are significantly different (*P* ≤ 0.05).
